# Endoplasmic reticulum stress and the unfolded protein response: emerging regulators in progression of traumatic brain injury

**DOI:** 10.1038/s41419-024-06515-x

**Published:** 2024-02-20

**Authors:** Yayi Yang, Dengfeng Lu, Menghan Wang, Guangjie Liu, Yun Feng, Yubo Ren, Xiaoou Sun, Zhouqing Chen, Zhong Wang

**Affiliations:** 1 Department of Neurosurgery & Brain and Nerve Research Laboratory, The First Affiliated Hospital of Soochow University, 188Shizi Street, Suzhou, 215006 Jiangsu Province China; 2 Suzhou Medical College of Soochow University, Suzhou, Jiangsu Province China

**Keywords:** Brain injuries, Molecular neuroscience

## Abstract

Traumatic brain injury (TBI) is a common trauma with high mortality and disability rates worldwide. However, the current management of this disease is still unsatisfactory. Therefore, it is necessary to investigate the pathophysiological mechanisms of TBI in depth to improve the treatment options. In recent decades, abundant evidence has highlighted the significance of endoplasmic reticulum stress (ERS) in advancing central nervous system (CNS) disorders, including TBI. ERS following TBI leads to the accumulation of unfolded proteins, initiating the unfolded protein response (UPR). Protein kinase RNA-like ER kinase (PERK), inositol-requiring protein 1 (IRE1), and activating transcription factor 6 (ATF6) are the three major pathways of UPR initiation that determine whether a cell survives or dies. This review focuses on the dual effects of ERS on TBI and discusses the underlying mechanisms. It is suggested that ERS may crosstalk with a series of molecular cascade responses, such as mitochondrial dysfunction, oxidative stress, neuroinflammation, autophagy, and cell death, and is thus involved in the progression of secondary injury after TBI. Hence, ERS is a promising candidate for the management of TBI.

## Facts

- The endoplasmic reticulum is highly sensitive to microenvironmental disturbances, and multiple factors can trigger its stress.
- The unfolded protein response can be activated by misfolded/unfolded proteins, and its three important initiation factors are the protein kinase RNA-like ER kinase, inositol-requiring protein 1, and activating transcription factor 6.
- Endoplasmic reticulum stress may be intertwined with a molecular cascade of responses such as mitochondrial dysfunction, oxidative stress, neuroinflammation, autophagy, and cell death, which together influence the progression of traumatic brain injury.
- Moderate endoplasmic reticulum stress can be protective, whereas chronic and severe stress may lead to deterioration.
- Interventions targeting endoplasmic reticulum stress are still emerging, although mainly in the preclinical stage.

## Open Questions

- How do multiple signaling pathways crosstalk with each other in the pathological progression of traumatic brain injury?
- How to determine the pro-survival and pro-apoptotic thresholds for endoplasmic reticulum stress after traumatic brain injury?
- How to develop endoplasmic reticulum stress-related molecular drugs in a targeted manner for optimal efficacy?

## Introduction

Traumatic brain injury (TBI) has a high incidence, lethality, and disability and seriously threatens people’s health worldwide [1]. Current management of TBI, including conservative and surgical treatment, is far from satisfactory. There is an urgent need for better therapies that address the mechanisms of TBI to help clinicians better cope with the disease. Over the decades, there has been increasing evidence that endoplasmic reticulum (ER) stress (ERS) is involved in the development of secondary injury after TBI through multiple pathways [2, 3]. However, a summary of the role and advances in research on ERS in TBI is rarely reported. Here, we summarize the progress of research in this area through an extensive review of the available evidence to provide new insight into the effective management of TBI.

## An overview of TBI

According to statistics, TBI has a global impact of 50–60 million people, costs the global economy approximately 400 billion dollars annually, and is expected to continue to rank among the top three causes of death and disability by 2030 [4]. TBI is an acute trauma caused mainly by motor vehicle crashes, falls, sports, and violent factors [5]. Specifically, TBI is also a chronic disease with adverse consequences over an extended period, potentially increasing the risk of neurodegeneration [6–9]. According to the Glasgow Coma Scale (GCS), the severity of impaired consciousness in patients with TBI can be classified as mild TBI (GCS 14-15), moderate TBI (GCS 9-13), and severe TBI (GCS 3–8) [10]. More than 90% of patients admitted were diagnosed with mild TBI [11]. To date, treatment options for TBI are often unsatisfactory. Mild TBI is mainly treated conservatively, while moderate to severe TBI may require both surgical and nonsurgical treatment depending on the contusion focus and intracranial pressure [12].

The progression of TBI can be summarized into two stages: primary injury and secondary injury (supplemental Fig. S1). When mechanical forces are strong enough to be transmitted to the brain, brain tissue along with cellular structures can be destroyed to cause primary injury, which includes cerebral contusions, lacerations, intracranial hematomas, diffuse axonal injuries, etc. [13]. After that, a series of molecular chain reactions lead to secondary injury [9]. Depending on the location and extent of the damage, focal or diffuse injuries may develop over a range of time from hours to years [14, 15], which may provide a potential time window for intervening in the prognosis of TBI.

The pathogenesis of secondary injury includes excitotoxicity, calcium overload, mitochondrial dysfunction, ERS, oxidative stress, neuroinflammation, and apoptosis, ultimately leading to pathological changes such as disruption of the blood-brain barrier (BBB), development of brain edema, increase in intracranial pressure, and delayed neurodegeneration [14, 16]. The extent of brain injury might be aggravated early or late in different stages of the events, resulting in deterioration. Exploring the mechanisms that lead to secondary injuries is particularly significant, as limited understanding makes clinical translation of basic research challenging. Considering the crosstalk with multiple pathogenic mechanisms and the critical involvement in TBI progression, a comprehensive review of the role of ERS in TBI was conducted based on available evidence.

## Signaling pathways of ERS

The ER is the leading site of protein synthesis and modification after translation [17] and is also involved in calcium ion storage and the metabolism of lipids and carbohydrates. As a reticular membrane structure, the ER can be sensitive to microenvironment disturbance. Many factors can activate ERS [18]. A rigorous quality control system helps ensure proper ER functioning and homeostasis maintenance. Under these sophisticated mechanisms, only mature proteins can travel to the cell surface or other organelles from the ER, while abnormal proteins are limited to the ER lumen or cytoplasm. Misfolded/unfolded proteins support cell survival by activating the unfolded protein response (UPR), which coordinates protein demand with the folding capacity to maintain intracellular equilibrium. Alternatively, the proteasome or autophagy can directly remove harmful proteins or polypeptides in ER-associated degradation (ERAD) [19–21]. When the intensity of ERS increases or persists for a prolonged period, cells may experience ER overload. At this point, the cell’s self-regulatory capacity is significantly impaired, followed by activation of the cell death program [22].

### ERS and UPR

**Fig. 1:**
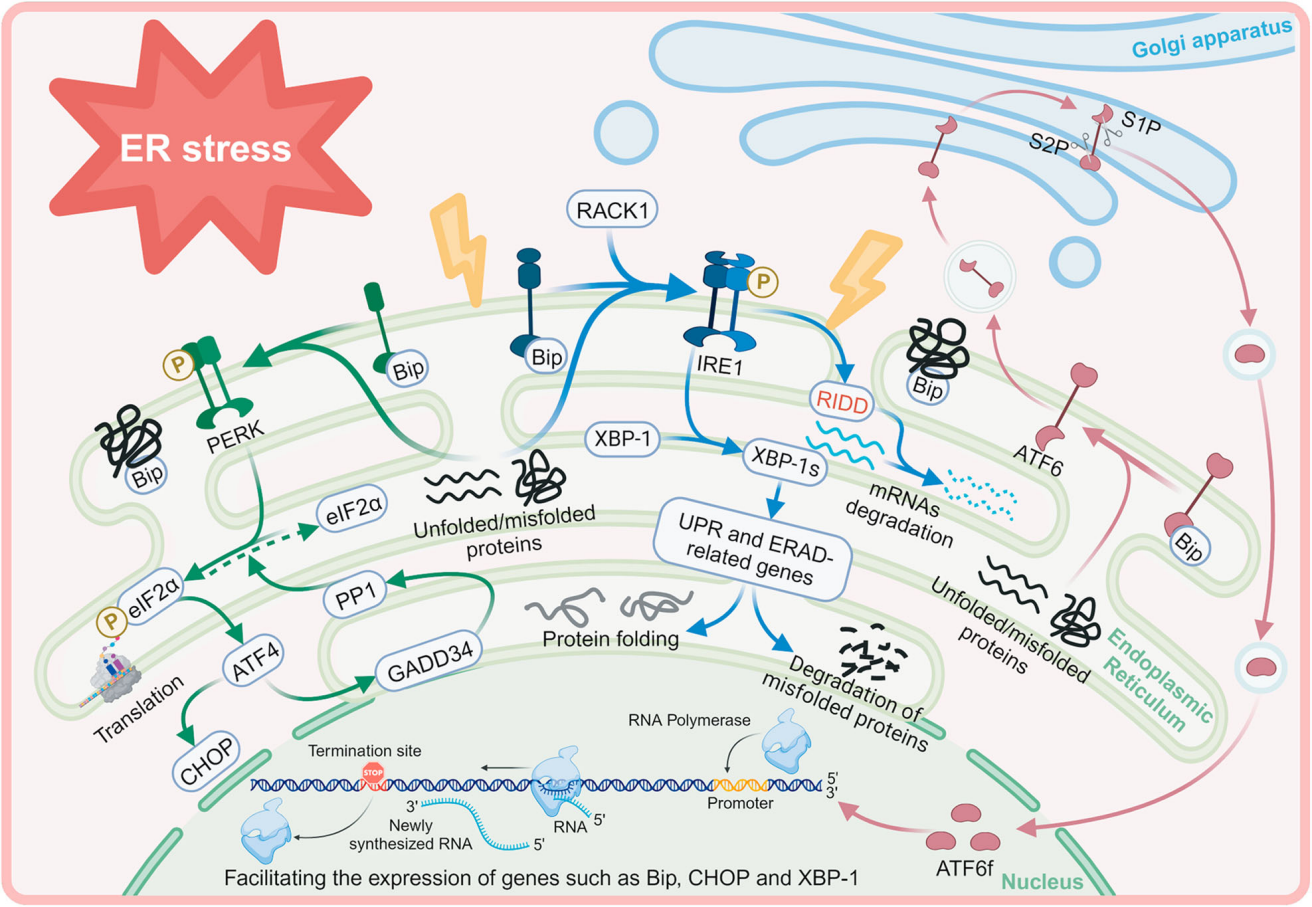
Schematic diagram of the ERS and UPR pathways. Created with BioRender.com. ER endoplasmic reticulum, UPR unfolded protein response, PERK protein kinase RNA-like ER kinase, eIF2α eukaryotic translation initiation factor 2 alpha, p-eIF2α: phosphorylated eIF2α, ATF4 activating transcription factor 4, PP1 type 1 protein phosphatase, GADD34 growth arrest and DNA damage-inducible gene 34, IRE1 inositol-requiring protein 1, XBP-1 X box binding protein 1, XBP-1s spliced XBP-1, RIDD regulated IRE1α‑dependent decay, ATF6 activating transcription factor 6, ATF6f fragment of ATF6α, ERAD ER-associated degradation, GLS Golgi-localization signal, S1P site-1 protease, S2P site-2 protease.

The UPR is considered a self-protection mechanism that coordinates transcription and translation between cells by monitoring the aggregation of misfolded/unfolded proteins [23–26]. Initiation of the UPR relies on three sensors: protein kinase RNA-like ER kinase (PERK), inositol-requiring protein 1 (IRE1), and activating transcription factor‑6 (ATF6) [27]. Under physiological conditions, these sensors combine with GRP78 (also called BiP) as a stable complex to maintain an inactive state. As misfolded/unfolded proteins increase, GRP78 and UPR sensors dissociate due to the high affinity of GRP78 and misfolded/unfolded proteins, thus stimulating downstream signal cascades (Fig. 1). The UPR may then promote survival or, in severe or chronic ERS, cell death [24, 28].

### PERK

PERK is a type I ER transmembrane protein with a cytoplasmic serine/threonine kinase structural domain. As a core component of the UPR, PERK regulates protein synthesis and translation and thus helps reduce the protein load in the ER [29, 30]. During the early stages of ERS, the accumulation of misfolded/unfolded proteins activates PERK, which further phosphorylates eukaryotic translation initiation factor 2 alpha (eIF2α) at Ser51. This inhibits the global production of protein [29, 31]. The promotion of specific translation of activating transcription factor-4 (ATF4) can be facilitated by phosphorylated eIF2α (p-eIF2α) [32]. As the primary target gene activator of ERS, the activation of ATF4 is dual under different conditions. During acute ERS, ATF4 collaborates with C/EBP-homologous protein (CHOP/GADD153) to decrease ER chaperone transcription to promote cellular adaptation and neuronal survival [33–35]. In contrast, ATF4 induces a proapoptotic program in the chronic phase by evoking aberrant signals that cause autophagy and inflammation [36]. Another downstream effector of ATF4 is GADD34, also known as PPP1R15A, which functions to counteract PERK activity by dephosphorylating eIF2α through protein phosphatase 1 (PP1) to reinitiate protein folding and translation [27, 37]. During ERS, selective inhibition of GADD34 exerts a protective effect by prolonging the phosphorylation of eIF2α [38, 39]. The delicate equilibrium between phosphorylation and dephosphorylation of eIF2α is crucial against ERS.

As a member of the eIF2α upstream kinase family, PERK exercises multiple biological functions and is, therefore, particularly important in neurodegeneration after TBI [40]. Interestingly, PERK may perform its function through alternative effectors. For example, one study reported that hindering the phosphorylation of PERK rescued spine loss and improved memory function after TBI [41]. This was attained by increasing phosphorylation of cyclic adenosine monophosphate response element binding protein (CREB) and postsynaptic density 95 (PSD95), which occurred through a process independent of p-eIF2α, suggesting that PERK may have novel substrates. Work by Farook and his colleagues [42] revealed that GADD34 competitively binds tumor necrosis factor (TNF) receptor-associated factor 6 (TRAF6) with Akt and induces cell death by inactivating Akt. Another study by Li et al. showed that JWH133, a selective cannabinoid receptor 2 agonist, facilitates the differentiation of microglia into the protective M2 phenotype and preservation of axons, thus alleviating white matter damage. This is achieved through a sequence of processes involving the downregulation of PERK, eIF2α, ATF4, and GADD34 expression, together with an upregulation of Akt phosphorylation [43].

In the context of TBI, the activation of PERK has also been found to stimulate the stimulator of interferon genes (STING) through phosphorylation. As a result, activated STING is vital in regulating microglial reactivity by targeting type I IFNs [44].

### IRE1

IRE1 is another type I ER transmembrane protein that encodes a duet of isoforms in the mammalian genome, namely, IRE1α and IRE1β. Like PERK, IRE1 comprises an active serine/threonine kinase structural domain and a ribonucleic acid endonuclease (RNase) structural domain. Most research studies have investigated the function of IRE1 [45], which possesses an ER luminal surface binding site for Bip/GRP78. Upon release of GRP78, IRE1 is activated by dimerization and autophosphorylation. IRE1 is then phosphorylated to acquire RNase activity and specifically splices the X-box binding protein (XBP-1) transcript into spliced XBP-1 (XBP-1s), making it a functional transcription factor [46]. XBP-1s may increase the transcription of several UPR- and ERAD-related genes, thereby enhancing ER folding and degradation to restore ER homeostasis [47, 48]. Once ERS is relieved, XBP-1s is replaced by unspliced XBP1 [49], indicating that the IRE1 signaling pathway functions against early ERS. The receptor for activated C kinase 1 (RACK1) activates IRE1 by inducing its autophosphorylation and is, therefore, one of the critical regulators of this sensor. Researchers have found that enhancing RACK1 alleviates neuronal apoptosis, BBB injury, and brain edema after TBI through the IRE1-XBP1 pathway, somewhat restoring neurological function [50].

Aside from targeting XBP1 during ERS, the RNase activity of IRE1 directly promotes the degradation of mRNAs through a distinct mechanism, regulated IRE1α‑dependent decay (RIDD) [51, 52]. Under physiological conditions, RIDD shows basic activity to participate in the maintenance of ER homeostasis. However, this activity may escalate during ERS, as observed in previous studies [52–55]. ER homeostasis equilibrium is restored only if the combination of XBP1s and RIDD can address the protein loads. When the accumulation of proteins cannot be mitigated, RIDD is activated to degrade mRNAs that encode pro-survival signals, resulting in cellular death [56, 57]. Therefore, although the IRE1 pathway initially promotes cell survival, it may still activate the RIDD pathway to trigger cell death if ERS persists. Research on the association between RIDD and TBI is currently limited. Nevertheless, it has been proven that IRE1 can trigger autophagy and apoptosis by recruiting various effectors to crosstalk with the nuclear factor-kappa B (NF-κB) and mitogen-activated protein kinase (MAPK) pathways in TBI [49].

### ATF6

PERK acts as a translational initiation suppressor, while IRE1 regulates the decay of mRNAs and the splicing of XBP1. These two branches alleviate the production load of proteins [58]. ATF6 likely belongs to the type II ER transmembrane proteins that strengthen the capacity to fold proteins. Typically, ATF6 is localized in the ER in non-stressed cells. As the Golgi-localization signal (GLS) on ATF6 is exposed under ERS, the entry of ATF6 into the Golgi complex becomes easier. ATF6 is sequentially cleaved by site-1 protease (S1P) and site-2 protease (S2P), releasing the ATF6f transcription factor and subsequently translocating to the nucleus [27]. ATF6f then promotes the activation of genes responsible for protein folding within the ER, such as GRP78, CHOP, and XBP-1. This transcriptional activation enhances the capacity of the ER to fold proteins [59, 60].

## ERS and mitochondrial disorder

**Fig. 2:**
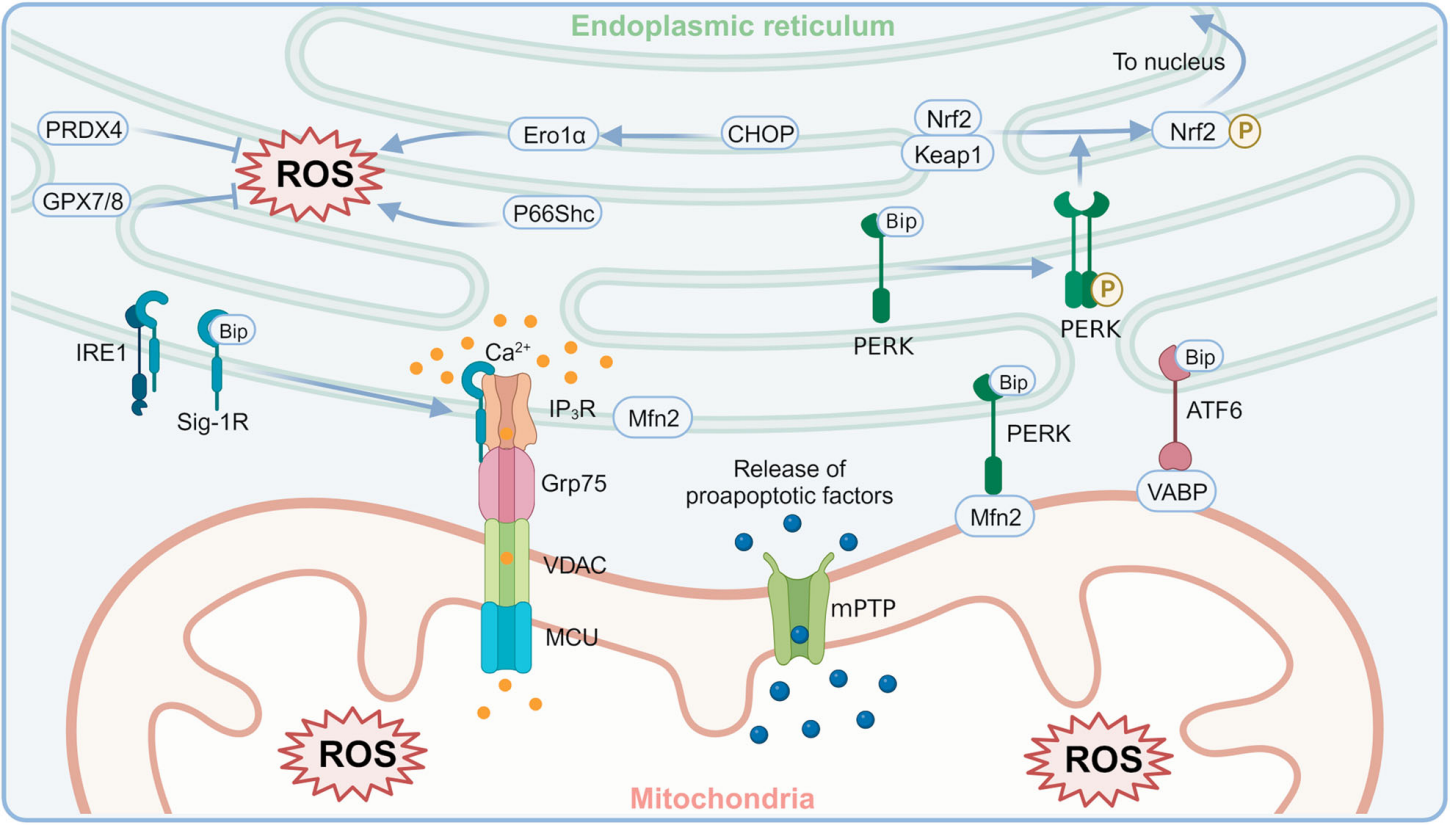
A schematic representation of ERS and mitochondrial dysfunction as well as oxidative stress. Created with BioRender.com. PERK protein kinase RNA-like ER kinase, Mfn2 mitofusin 2, Nrf2 nuclear factor erythroid 2-related factor 2, Keap1 Kelch-like ECH-associated protein 1, IRE1 inositol-requiring protein 1, Sig-1R sigma-1 receptor, IP_3_R inositol trisphosphate receptor, Grp75 75 kDa glucose-regulated protein, VDAC voltage-dependent anion channel, MCU mitochondrial calcium uniporter, ATF6 activating transcription factor 6, VABP recombinant vesicle-associated membrane protein-associated protein B, CHOP C/EBP-homologous protein, Ero1α ER oxidase 1 alpha, ROS reactive oxygen species, PRDX4 peroxiredoxin 4, GPX7 glutathione peroxidase 7, GPX8 glutathione peroxidase 8, mPTP mitochondrial permeability transition pore.

Mitochondria are another highly dynamic organelle in mammalian cells. Healthy mitochondria continuously produce ATP to provide energy for various biological processes, while metabolic byproducts such as proteins, DNA, RNA, reactive oxygen species (ROS), and lipids are generated [61]. The dynamic mitochondrial-associated membrane (MAM) establishes the physical connection between mitochondria and the ER (Fig. 2) [62]. It enables the transfer of information and materials between the two organelles, thereby ensuring cell viability [63, 64]. Moreover, the increased contact between the two organelles facilitates the initiation and transmission of ERS signaling. This process induces the reorganization of the ER and mitochondrial networks [65]. Studies indicate that MAMs are involved in critical biological processes such as Ca^2+^ homeostasis, mitochondrial dysfunction, ERS, lipid metabolism, inflammatory response, autophagy, and apoptosis [66–68].

The ER is one of the critical intracellular calcium reservoirs, and MAM provides a pathway for ER-mitochondrial Ca^2+^ transfer [69]. There is a dynamic chaperone protein called the Sigma-1 receptor (Sig-1R) on MAMs, which serves as a scaffold with multiple functions; this benefits ER-mitochondrial crosstalk [70]. Sig-1R and GRP78 form a stable complex under physiological conditions. In response to stress, Sig-1R disengages from Grp78 and binds to the inositol trisphosphate receptor (IP_3_R), allowing the transfer of excess Ca^2+^ from the ER to mitochondria via the IP_3_R-Grp75-VDAC complex [71]. Mitochondrial Ca^2+^ overload impairs the mitochondrial electron transport chain and leads to peroxide accumulation. This increased mitochondrial calcium load makes mitochondria more vulnerable to ROS, which may activate the mitochondrial permeability transition pore (mPTP), the release of proapoptotic factors, and finally, apoptosis [72–74]. Additionally, Ca^2+^ can be regulated by Mfn2, which modulates ERS and mitochondrial dysfunction through PERK [75]. The interaction between PERK in MAM and Mfn2 is essential for maintaining strict ER-mitochondrial contacts and mediating mitochondrial ROS-mediated apoptosis [76]. The depletion of Mfn2 triggers the activation of PERK, which induces ERS [77]. In addition to PERK, two other sensors initiating the UPR are also present on the MAM. IRE1 is involved in regulating Ca^2+^ transfer and ATP production through IP_3_R [78], and its ubiquitination prevents ERS-induced apoptosis [79] Furthermore, ATF6 directly inhibits the UPR response by interacting with the tethering protein VAPB [80] and exerts a pro-survival effect by regulating Ca^2+^ transfer in MAMs [81].

Considerable attention has been given to the pathophysiological link between MAM and neurological disorders. A preliminary summary of the potential involvement of MAM in TBI was presented by Sun et al. in 2017 [67]. Sig-1R is detected in various cells in the central nervous system (CNS) and has been implicated in several neurological disorders, including TBI [82–85], Alzheimer’s disease (AD) [86], Huntington’s disease (HD) [87], Parkinson’s disease (PD) [88], and amyotrophic lateral sclerosis (ALS) [89]. It has been demonstrated that Sig-1R facilitates cellular calcium homeostasis, excitability regulation, and ROS clearance. Furthermore, it may affect various pathophysiological processes after TBI by stabilizing the function of the MAM, ER, and mitochondria [82]. Sig-1R regulates neuroinflammation mediated by astrocytes and microglia [85, 90–93], recognized as a hallmark event of secondary brain injury after TBI [94–97]. Tan et al. found that ERS was suppressed, and mitochondrial function was restored in TBI rats pretreated with salubrinal (SAL), a well-recognized ERS inhibitor [98]. In addition, Li et al. showed that polydatin could prevent mitochondrial apoptosis by increasing silent information regulator family protein 1 (SIRT1) expression and has a neuroprotective effect by inhibiting proteins such as p-PERK, XBP-1(s), and cleaved ATF6 [99]. All the studies above suggest that ERS and mitochondrial function are inextricably connected during the development of TBI. Further studies are needed to clarify the above compounds’ role in ERS and expand their applications in TBI.

## ERS and oxidative stress

Under physiological conditions, small-scale ROS can be produced in several membranous organelles, such as the ER and mitochondria [100]. However, when the balance between generating and scavenging oxygen-derived free radicals is disturbed, oxidative stress arises [101]. This process triggers a variety of signaling pathways, such as PI3K/Akt, MAPK, nuclear factor erythroid 2-related factor 2 (Nrf2)/Keap1, NF-κB, and p53, to participate in cell survival or death processes [102, 103].

Disulfide bond formation is a critical protein folding step to form a spatial conformation. The catalysis of this process relies on ER oxidase 1α (Ero1α), which undergoes electron transfer between Ero1α itself and molecular oxygen to produce the byproduct H_2_O_2_, a ROS [21, 104]. High expression of Ero1α is closely associated with an increase in ROS [105–109].

In the TBI context, oxidative stress and ERS are closely related and they interact with each other to promote the pathological process. For example, a series of modifications of the 66 Kd Shc protein (p66Shc) (including phosphorylation, heterodimerization, etc.) and its translocation at MAM can promote ROS production [110, 111]. Moreover, enzymes such as peroxiredoxin 4 (PRDX4), glutathione peroxidase 7 (GPX7), and GPX8 within the ER scavenge H_2_O_2_ to exert antioxidants and maintain ER homeostasis [112, 113].

When exposed to oxidative stress, cells may activate the unfolded protein response (UPR), which in turn disrupts the normal formation of disulfide bonds in the ER, induces the expression of UPR genes such as CHOP, and exacerbates oxidative stress [21, 114, 115]. Nrf2 is considered to regulate the cellular antioxidant response [116]. Under physiological conditions, Nrf2 remains inactive in combination with Keap1. Upon PERK-dependent phosphorylation of Nrf2, the Nrf2/Keap1 complex is disrupted, allowing Nrf2 to translocate to the nucleus to promote the expression of antioxidant genes [117]. Together with ATF4, Nrf2 can induce antioxidant response element (ARE)-dependent gene transcription [118]. Additionally, proapoptotic CHOP triggers the expression of Ero1α to exacerbate ER oxidation [119]. As a stress-inducible protein, Sestrin2 maintains intracellular homeostasis through multiple signaling pathways. It has been shown that Sestrin2 can be activated by ATF4, Nrf2, and C/EBP-β of the PERK pathway, and subsequently exerts anti-oxidative stress and anti-apoptosis effects through the adenosine monophosphate (AMP)-activated protein kinase (AMPK)/mechanistic target of rapamycin complex 1 (mTORC1) pathway [120]. Thus, the PERK signaling pathway tightly links ERS to oxidative stress [117, 121]. ERS and oxidative stress coexist and enhance each other in multiple pathological states (Fig. 2), but the specific mechanisms remain to be further elucidated.

Liu et al. showed that overexpression of Sestrin2 attenuated oxidative stress and apoptosis in in vivo and in vitro models and ameliorated cerebral edema and neurological deficits in TBI mice by enhancing Nrf2 signaling [122]. In a study based on the blast TBI model, the authors claimed that SAL administration significantly reduced CHOP activation, decreasing the nicotinamide adenine dinucleotide phosphate (NADPH) oxidase 4 (NOX4) activation (mediating ROS production) and reducing the products of oxidative stress. It suggests that targeting ERS can diminish oxidative stress and alleviate secondary injury after TBI [123]. Likewise, the neuroprotective effects of naringenin have been demonstrated in several neurological disorders. For example, Deng et al. revealed that continuous administration of naringenin inhibited ER stress (p-eIF4α, ATF3, and CHOP), oxidative stress, and apoptosis after TBI [124]. Moreover, another clinical drug study confirmed the role of combined antioxidant and anti-ERS therapies in TBI [125]. It showed that the combination of apocynin (reducing ROS formation), tert-butylhydroquinone (promoting ROS disposition), and SAL (preventing ERS) not only reduced oxidative DNA damage and inflammatory cell infiltration but also reduced lesion volume and neurobehavioral deficits. It is thus evident that oxidative stress after TBI is closely associated with ERS and that interventions targeting both pathological processes are likely to effectively limit secondary damage after TBI.

## ERS and cell death in TBI

Neuronal cell death occurs in various forms [126]. This discussion focuses on the correlation between programmed cell death (PCD) and ERS in TBI. PCD is vital to maintaining homeostasis by eliminating damaged or diseased cells; it is mainly controlled by genes and is an autonomous and systematic cell death process. PCD has two primary types: apoptosis (type I) and autophagic cell death (ACD, which is type II). Among them, apoptosis is categorized into two primary pathways: the extrinsic/death receptor pathway and the intrinsic/mitochondrial pathway [35]. Both pathways are interconnected by ER-induced apoptosis [127]. When intracellular Ca^2+^ overloads, post-apoptotic neurons undergo secondary necrosis, stimulating inflammatory responses [128]. The autophagic process comprises four steps: the induction and formation of a phagosome, the fusion of the autophagosome and lysosome, and ultimately, degradation and recycling. The degree of autophagy is a critical factor in determining the outcome of TBI [129].

### ERS and apoptosis in TBI

**Fig. 3:**
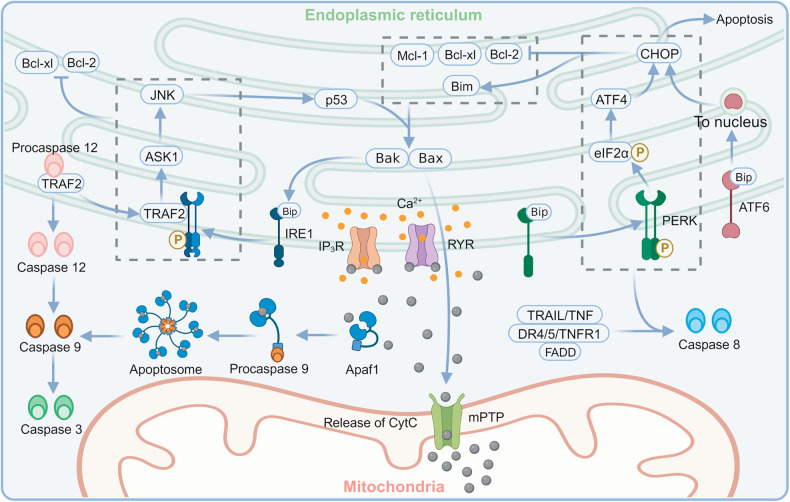
Schematic diagram of ERS-induced apoptosis. Created with BioRender.com. Bcl-xl B-cell lymphoma-extra large, Bcl-2 B-cell lymphoma 2, Bak Bcl-2 homologous antagonist-killer protein, Bax Bcl-2-associated X protein, Bim Bcl-2 interacting mediator of cell death, Mcl-1 myeloid cell leukemia sequence 1 protein, TNF tumor necrosis factor, TRAIL TNF-related apoptosis-inducing ligand, TNFR1 tumor necrosis factor receptor 1, TRAF2 tumor necrosis factor receptor-associated factor 2, DR death receptor, FADD Fas-associated death domain, IRE1 inositol-requiring protein 1, ASK1 apoptosis signal-regulating kinase 1, JNK c-Jun N-terminal kinase, PERK protein kinase RNA-like ER kinase, eIF2α eukaryotic translation initiation factor 2 alpha, ATF activating transcription factor, CHOP C/EBP-homologous protein, IP_3_R inositol trisphosphate receptor, RYR ryanodine receptor, mPTP mitochondrial permeability transition pore, CytC cytochrome c.

To alleviate ERS, the UPR lowers the levels of misfolded/unfolded proteins. The related degradation pathway increases the expression of folding chaperones and protein hydrolases while inhibiting translation to decrease the demand for protein folding. However, apoptosis is initiated if these pathways fail to restore protein homeostasis (Fig. 3) [130, 131]. Secondary injury in TBI is often characterized by apoptosis, and its inhibition may be beneficial in reducing functional deficits and potentially restoring lost functions in TBI patients.

#### ERS-induced intrinsic/mitochondrial apoptosis

ERS-induced apoptosis predominantly occurs through the intrinsic/mitochondrial pathway [132, 133] and is carried out through mPTP opening. The antiapoptotic Bcl-2 family suppresses the opening of mPTP, while the proapoptotic BH3-only proteins (with Puma, Noxa, and Bim included) activate Bax/Bak to induce mPTP formation and cytochrome c (Cytc) release [127]. Generally, the IRE1/apoptosis signal-regulating kinase 1 (ASK1)/ c-Jun N-terminal kinase (JNK) pathway and PERK/ATF4/CHOP are crucial pathways that mediate ERS-induced apoptosis [26, 134].

IRE1 activation separates TNF receptor-associated factor 2 (TRAF2) from procaspase12 on the ER membrane, activating ASK1, a widely expressed mitogen-activated protein kinase kinase kinase (MAPKKK). This triggers the JNK pathway, followed by activation of p53 and upregulation of Bax. This process increases mitochondrial membrane permeability, releases Cytc into the cytoplasm, and activates apoptotic protease-activating factor 1 (Apaf1)-dependent caspase [135]. When Cytc binds to Ca^2+^ channels on the ER membrane, such as IP_3_R and ryanodine receptor (RYR), the release of Ca^2+^ is initiated. After separation from TRAF2, procaspase12 dimerizes and translocates to the cytoplasm, which leads to the activation of caspase9. In addition, Cytc can directly activate caspase9, which subsequently activates caspase3 to cause apoptosis [135, 136]. A recent study by Ghosh et al. showed that the IRE1/ASK1/JNK pathway might result in the inactivation of Bcl-2 and Bcl-xl through phosphorylation. They also discovered that a small molecule metabotropic inhibitor, KIRA6, successfully reduced ERS-related apoptosis by inhibiting IRE1 activation [137]. Another study indicated that IRE1 directly interacted with Bax/Bak to activate downstream signaling [138].

Apart from IRE1, the other two UPR sensors can also regulate CHOP transcription upstream. ATF6 promotes the expression of CHOP and triggers apoptosis upon being sheared to ATF6f into the nucleus [139, 140]. In contrast, PERK activation leads to eIF2α phosphorylation, which induces ATF4 expression and activates CHOP. CHOP can downregulate Bcl-2 family proteins and upregulate Bim. Since Bim is a BH3-only protein that mediates Bax/Bak, the activation of CHOP can lead to enhanced outer membrane permeability, ultimately resulting in the release of Cytc and the caspase cascade [141].

#### ERS-induced extrinsic/death receptor apoptosis

Extrinsic/death receptor apoptosis involves the interaction between death ligands (such as TRAIL, TNF, or Fas ligands) and the corresponding receptors (TRAIL-R1/DR4, TRAIL-R2/DR5, TNFR1, and Fas). This leads to the recruitment of the Fas-associated death domain (FADD) adapter protein, thus inducing caspase8-dependent apoptosis. A study showed that the PERK/ATF4/CHOP pathway could upregulate DR5 and activate caspase8 via TRAIL [142]. Additionally, it was reported that CHOP could bind DR4 directly or indirectly to participate in extrinsic/death receptor apoptosis [143]. Several animal experiments have investigated the effects of ERS-related pathways on secondary injury following TBI (Table 1). Such evidence suggests that inhibition of ERS in multiple ways may significantly reduce TBI-induced apoptosis.

**Table 1:** Studies of ERS-related pathways in the TBI models.

Study	Interventions	Models& animals	Changes in relevant markers	Main effects
Hood et al. [211]	Knocking out CHOP	CCI mice	Significantly increasing the number of ipsilateral doublecortin-positive cells	Knocking out CHOP led to a decrease in neonatal neuronal damage
Begum et al. [214]	DHA	CCI rats	Downregulating eIF2α, ATF4, IRE1 and CHOP; Minimizing the buildup of ubiquitinated proteins and APP/p-Tau	DHA administration lowered the level of ERS, reduced the accumulation of abnormal proteins, and contributed to the early restoration of sensory function
Lucke-Wold et al. [215]	DHA	Blast TBI rats	Reducing tau hyperphosphorylation and the activation of CHOP	DHA reduced ERS and improved cognitive function in rats
Liu et al. [2]	Knocking out PI3Kγ	CCI mice	Downregulating PERK/ATF4/CHOP; Reducing the death of neurons	Induction of neuronal PI3Kγ contributed to the development of chronic ERS, triggered neuronal apoptosis, and resulted in long-term functional impairment of neurons
Deng et al. [124]	Naringenin	Weight-drop mice	Increasing the expression of p-eIF2α and Bcl-2; Reducing the expression of ATF4, GRP78, CHOP and Bax; Reducing the number of TUNEL-positive cells	Naringenin inhibited TBI-induced activation of ERS pathway, oxidative stress, and apoptosis
Sun et al. [216]	Dexmedetomidine	CCI mice	Reducing the expression of p-PERK, p-eIF2α, ATF4, CHOP, Bax, p-JNK, and caspase12; Reducing the number of TUNEL-positive neurons	Dexmedetomidine significantly reduced TBI-induced ERS and ERS-related neuronal apoptosis, reduced brain edema, and improved neurological functions
Wang et al. [217]	Salubrinal	Weight-drop mice	Upregulating p-eIF2α, Bcl-2; Downregulating GRP78, ATF4, CHOP, caspase12 and cleaved caspase3; Reducing TUNEL-positive cells in quantity	Salubrinal treatment improved morphological and functional outcomes in TBI-induced mice, and these neuroprotective effects might be related to the inhibition of apoptosis, at least partially through the inhibition of the ERS-related autophagy pathway
Logsdon et al. [218]	Salubrinal	Blast TBI rats	Reducing the expression of CHOP and cleaved caspase3	Salubrinal inhibited ER-related apoptosis and ameliorated blast TBI-induced impulsive behavior
Gao et al. [201]	Salubrinal	Weight-drop mice	Reducing the expression of GRP78	IL-33/ST2 attenuated brain edema and recovered motor and cognitive function partially by inhibiting autophagy, ERS, apoptosis, and neuroinflammation
Wang et al. [219]	Mild hypothermia	CCI mice	Reducing the expression of ERS markers: p-eIF2α/eIF2α, ATF4, CHOP and IRE1; Reducing the nuclear translocation of CHOP	Mild hypothermia reduced ERS-induced apoptosis and improved neuronal function after severe TBI
Chen et al. [220]	Knockdown of Arc expression with Si-Arc-3	Modified Mukhin’s TNI on cultured neurons	Increasing the expression of Grp78, CHOP and caspase12	Arc silence exacerbated neuronal death by upregulating ER-related apoptosis and necrosis
Sun et al. [221]	TUDCA with Akt inhibitor	CCI mice	Inhibiting PERK/ATF4/CHOP and caspase12; Increasing p-Akt expression and the ratio of Bcl-2/Bax	TUDCA might act as an ERS inhibitor to attenuate apoptosis and activate the Akt pathway to alleviate brain edema to improve neurological function
Sun et al. [222]		FPI rats	Increasing expression of Grp78, caspase12 and caspase3 in the traumatic penumbra	ERS-related apoptosis was activated in the penumbra and might affect the process of secondary injury
Wu et al. [223]	Vitamin B12	CCI mice	Downregulating GRP78, IRE1, XBP-1, and CHOP; Reducing GRP78 positive cells in quantity; Increasing the expression of tau protein	Vitamin B12 ameliorateed nerve injury after TBI by downregulating caspase12-induced apoptotic pathways and reversed the upward trend of ERS-related proteins; Vitamin B12 had the same efficacy as the ERS inhibitor 4-PBA in regenerating axon.

*CHOP*: C/EBP-homologous protein, *DHA* docosahexaenoic acid, *PI3Kγ* phosphoinositide 3-kinase gamma, *Si-Arc-3* Arc expression using small interfering RNA, *TUDCA* taurine ursodeoxycholic acid, *Akt* protein kinase B, *p-Akt* phosphorylated Akt, *PERK* protein kinase RNA-like ER kinase, *p-PERK* phosphorylated PERK, *eIF2α* eukaryotic translation initiation factor 2 alpha, *p-eIF2α* phosphorylated eIF2α, *ATF* activating transcription factor, *IRE1* inositol-requiring protein 1, *XBP-1* X-box binding protein 1, *APP* amyloid precursor protein, *p-Tau* phosphorylated tau, *Bcl-2* B-cell lymphoma 2, *Bax* Bcl-2-associated X protein, *GRP78* glucose-regulated protein 78, *TUNEL* terminal deoxynucleotidyl transferase dUTP nick-end labeling, *JNK* c-Jun N-terminal kinase, *p-JNK* phosphorylated JNK, *CCI* controlled cortical impact, *FPI* fluid percussion injury, *bTBI* blast-induced traumatic brain injury, *TNI* traumatic neuronal injury, *ERS* endoplasmic reticulum stress, *IL-33* interleukin-33, *ST2* growth STimulation expressed gene 2, *4-PBA* 4-phenyl-butyric acid.

### ERS and autophagy in TBI

Autophagy is a self-defense mechanism of eukaryotic cells against stress mediated by lysosomes to degrade damaged organelles and misfolded/unfolded proteins to recycle the resulting macromolecules to produce new proteins and organelles [144–146]. However, excessive activation or inhibition of autophagy leads to disruptions in brain proteostasis, which is critical to maintaining normal neuronal function [147]. Autophagic flux plays a crucial role in degrading abnormal proteins and organelles. Impaired autophagic flux leads to intracellular accumulation rather than elimination of abnormal proteins or organelles [129]. In addition to lysosomal function [148], ERS is also a significant factor affecting autophagic flux in TBI [3]. ERS activates autophagy to degrade misfolded/unfolded proteins when ERAD cannot address it [149]; conversely, impaired UPR or autophagy results in extensive cell death [149, 150]. Notably, the role of autophagy in TBI is a matter of debate. The varying degrees of autophagy may have opposing effects on TBI [151], depending on the severity and type of brain injury and the timing of autophagy induction [151, 152]. Autophagy induction aims to provide neuroprotective effects when the autophagic flux is not disrupted, as seen in a study based on a cerebellar TBI model [153]. However, impaired autophagy and autophagic flux may instead promote cell death [153]. Furthermore, another team suggested that ERS-induced autophagy promoted neurological dysfunction after TBI through the ATF6 signaling pathway, while its pharmacological blockade alleviated TBI-induced secondary brain injury [3]. As such, the role of autophagy in TBI remains to be elucidated by additional research.

## ERS and neuroinflammation in TBI

Neuroinflammation is a complex response mechanism involving a cascade of interactions between cellular components, cytokines, and chemokines in the CNS. This response is crucial for immune defense against pathological stimuli. However, its abnormal activation is also one of the critical drivers of secondary injury after TBI, and may therefore have both positive and negative effects on the pathological progression of TBI [94, 154]. Once ERS occurs, the UPR and its downstream components engage in complex crosstalk with inflammatory pathways such as NF-κB, Janus kinase/signal transducer and activator of transcription (JAK/STAT), and MAPK [155]. These pathways are crucial for the fate of neurons, microglia, astrocytes, etc.

### NF-κB signaling pathway

In the human brain, NF-κB typically exists as a p65/p50 dimer and is maintained in a state of rest by binding with unphosphorylated κB inhibitors (IκB). Upon phosphorylation of IκB by IκB kinase (IKK), NF-κB/IκB depolymerizes, allowing free NF-κB to relocate from the cytoplasm to the nucleus, subsequently initiating downstream signaling cascades [156].

NF-κB regulates neuroinflammation by regulating the expression of genes that encode chemokines and inducible nitric oxide synthase (iNOS) [157]. Furthermore, NF-κB contributes to astrocyte swelling (cytotoxic edema) and serves as a downstream component in post-TBI mammals that can be activated by various receptors, including TNF receptor-associated factor 6 (TRAF-6) and Toll-like receptor 4 (TLR-4) [158–160]. Following TBI, increased NF-κB expression in glial cells exacerbates neuroinflammation [161, 162]. Moreover, NF-κB is also involved in synaptic plasticity [163, 164], and abnormal activation of this pathway may cause synaptic dysfunction, leading to cognitive decline [165]. Hence, it is promising to attenuate secondary injury after TBI by targeting the NF-κB pathway. Deng et al. reported that translation inhibition, mediated by eIF2α phosphorylation following ERS, could be instrumental in NF-κB activation [166]. Another study indicated that NF-κB induced by ERS activation was impaired in IRE1 knockdown cells [167]. An in vitro study by Yamazaki et al. showed that inhibiting ATF6, rather than PERK or IRE1, also prevented the activation of NF-κB [168]. In this light, all three pathways of the UPR seem to be closely associated with NF-κB. As mentioned previously, inhibition of NF-κB could be a possible way to reduce neuroinflammation after injury. Credible evidence suggests that the same effect may be exerted by inhibiting the three branches of the UPR.

### JAK/STAT signaling pathway

Immune cells are first recruited to the injury site, and then the JAK/STAT pathway is activated to mediate the inflammatory cascade response. It is crucial for initiating innate immunity, coordinating adaptive immunity, and constraining inflammation and the immune response [169–171]. In addition, the JAK/STAT pathway is also responsible for delivering cytokines and growth factors involved in various biological processes [172]. Upon binding to specific ligands, JAK initiates the phosphorylation and dimerization of STAT. This leads to the translocation of STAT into the nucleus, where it binds to DNA sequences and regulates gene expression [172]. In astrocytes, this pathway can be activated by PERK, thus inducing an inflammatory response and further stimulating microglial activation, as demonstrated in a mouse model of multiple sclerosis (MS) [173]. In melanoma, JAK/STAT can be enhanced by ectopic expression of IRE1 or XBP1s in response to the proinflammatory cytokine IL-6, thus promoting tumor cell proliferation [174]. These studies suggest that the UPR can regulate the JAK/STAT pathway in various ways [169, 173, 175].

The available evidence has shown that activation of the JAK/STAT pathway has extensive involvement in multiple pathological processes such as inflammatory cascade [176], BBB disruption [177, 178], and axonal regeneration [179] after TBI. However, the interaction of the pathway with ERS after TBI has not been reported.

### MAPK pathway

MAPKs are conserved serine-threonine kinases consisting of four subfamilies: ERK, p38, JNK, and ERK5 [180]. Crosstalk between MAPKs and the UPR has been extensively studied [181–183], and knowledge of this mechanism helps to deepen our understanding of ERS after TBI. Multiple members of the MAPK family can be stimulated and activated by one or more UPR pathways, leading to the development or progression of neuroinflammation [184] and apoptosis [185] by increasing the production of inflammatory cytokines. For example, PERK and ATF6 activate ERK1/2 and p38, while all three UPR pathways induce JNK activation [186]. The IRE1-dependent activation of ERK1/2 and JNK after ERS helps promote cell survival and axonal regeneration [187, 188]. Moreover, the p38-dependent phosphorylation of CHOP and ATF6 is essential for the MAPK signaling pathway to regulate the UPR and ultimately promote apoptosis [189–191]. In a mouse model of polycystic ovary syndrome (PCOS), metformin mitigated the deleterious effects of UPR activation by decreasing p38 MAPK phosphorylation in ovarian granulosa cells (GCs) [192]. It was revealed that ERS triggers the activation of p38 MAPK on lysosomes through PERK-dependent mitogen-activated protein kinase kinase 4 (MKK4). Subsequently, phosphorylation of p38 results in the activation of chaperone-mediated autophagy (CMA) [193, 194]. This process may be critical for promoting the survival of SNc dopaminergic neurons in PD mice [195].

The involvement of the MAPK pathway in neuroinflammation after TBI is widely supported by evidence. Inhibiting the p38 MAPK pathway with SIRT1 [196], curcumin [197], and specific doses of human mesenchymal stem cell-derived extracellular vesicles (hMSC-EVs) [198] may help limit the progression of neuroinflammation after TBI. Several studies have suggested an association between ERS and neuroinflammation in TBI. In 2015, Harvey and his colleagues [199] revealed that microglia or macrophages activated after TBI were spatially associated with CHOP-positive neurons. It was found that administering docosahexaenoic acid (DHA) simultaneously reduced ERS, NF-κB activation, and microglia or macrophage polarization. This implies that there may be a correlation between ERS and neuroinflammation following TBI. It was reported that IL-33 acts on microglia/macrophages to regulate neuroinflammation [200]. The work by Gao et al. in 2018 [201] suggested that SAL alone or combined with IL-33 prevented ERS, autophagy, and apoptosis by inhibiting the increase in IL-1β and TNF-α caused by TBI. Another study [2] revealed a potential association between neuroinflammation and ERS after TBI. Phosphoinositide 3-kinase γ (PI3Kγ) was reported to be related to neuroinflammation in several neurological disorders [202–205]. Following TBI, PI3Kγ expression in the cortex and hippocampus might spatiotemporally coincide with the activation of neuronal ERS pathways (p-eIF2α, ATF4, and CHOP). Moreover, the deletion of PI3Kγ significantly attenuated the activation of ERS pathways and neuronal cell death post-TBI. Given the intricate pathophysiology of TBI, a combination of pharmacological interventions may be able to produce synergistic effects. Recent research [125] based on the controlled cortical impact (CCI) mouse model demonstrated the efficacy of combination therapy involving apocynin, tert-butylhydroquinone, and SAL. It was found to suppress the posttraumatic inflammatory response by decreasing T-cell and neutrophil infiltration and microglia activation while alleviating oxidative DNA damage. The therapeutic potential of triple therapy was confirmed by reducing oxidative stress, ERS, and neuroinflammation. In summary, although limited, the existing evidence may provide some illuminating implications for future exploration of mechanisms targeting cytotoxic proteins and neuroinflammation after TBI.

## ERS in TBI pathology

**Fig. 4:**
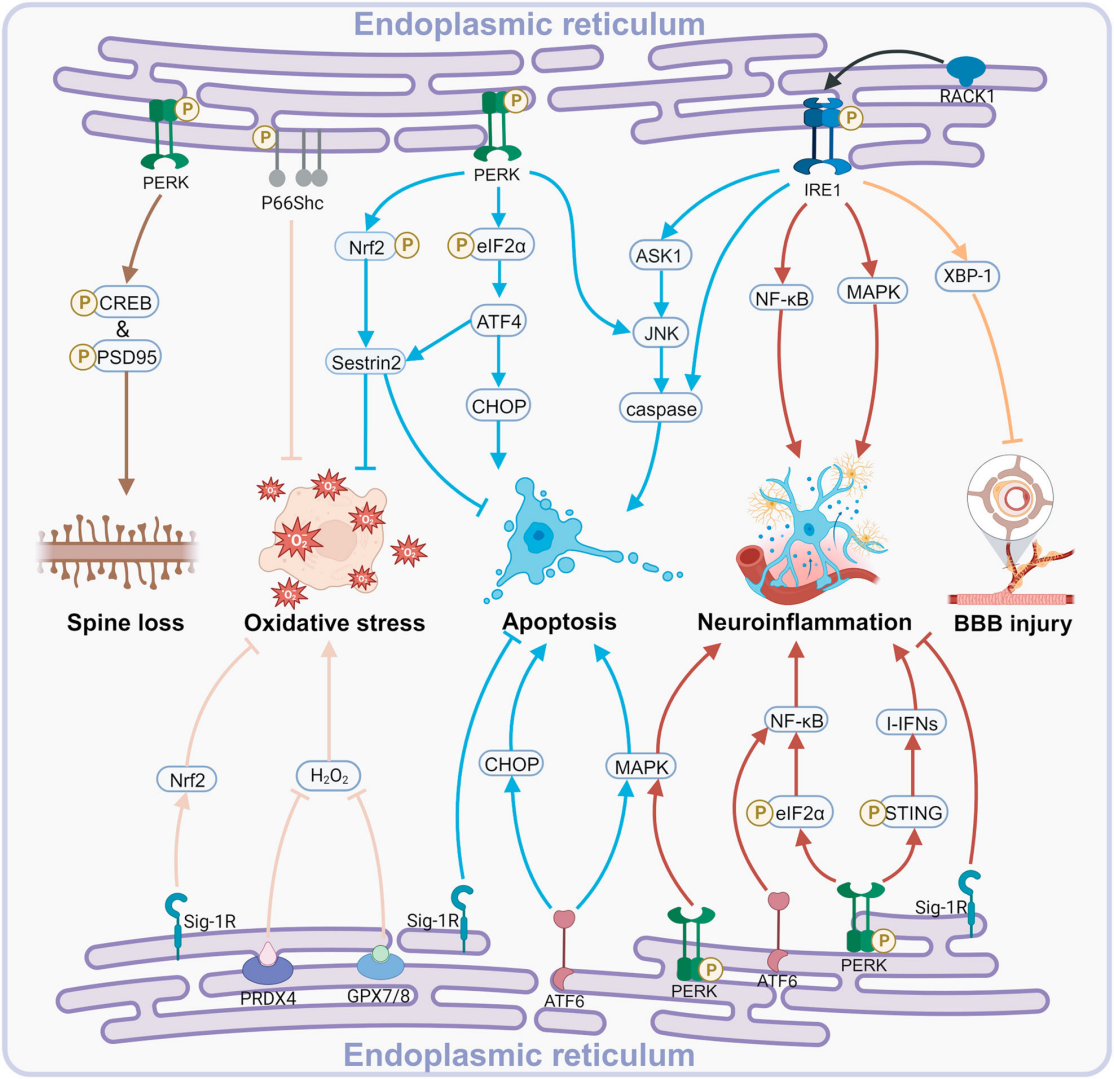
The intricate association between ERS and various pathologies after TBI. Created with BioRender.com. PERK protein kinase RNA-like ER kinase, CREB cyclic adenosine monophosphate response element binding protein, PSD95 postsynaptic density 95, Sig-1R Sigma-1 receptor, P66Shc the 66 Kd Shc protein, Nrf2 nuclear factor erythroid 2-related factor 2, PRDX4 peroxiredoxin 4, GPX7/8 glutathione peroxidase 7/8, eIF2α eukaryotic translation initiation factor 2 alpha, ATF4/6 activating transcription factor4/6, CHOP C/EBP-homologous protein, ASK1 apoptosis signal-regulating kinase 1, JNK c-Jun N-terminal kinase, MAPK mitogen-activated protein kinase, NF-κB nuclear factor-kappa B, I-IFN type I interferon, STING stimulator of interferon genes, XBP-1 X-box binding protein.

Clinical TBI is a highly heterogeneous injury, and sources of this heterogeneity include the site of injury, the nature of the causative force, and the severity of the injury [206, 207]. Despite the many controllable variables in preclinical TBI models, the heterogeneity is still not negligible due to the diversity of combinations of impact parameters in multiple models [208]. Nevertheless, ERS, oxidative stress, neuroinflammation, BBB injury, and various patterns of death, including apoptosis, are pathologies common to multiple TBIs. Figure 4 demonstrates the understanding of the intricate link between UPR branches and pathologic progression after TBI. As can be seen, ERS is closely associated with oxidative stress, neuroinflammation, and apoptosis after TBI.

In addition, TBI is usually caused by sudden violence, which is very different from neurodegenerative diseases and stroke. When the causative factor is too strong (e.g., severe TBI), which has been reported to be distinct from mild to moderate TBI [209, 210], it remains unknown whether the UPR is protective or death-promoting in such cases. Focal and diffuse TBI are also an important source of heterogeneity [208]. The common unilateral CCI, lateral fluid percussion (LFP), and weight drop (WD) models simulate clinically focal injuries, where the injury is mainly confined to the cortex and can spread to subcortical structures. Midline fluid percussion injury (mFPI) and blast TBI, which simulate diffuse injuries, tend to injure subcortical structures, such as the up and down conduction bundles of the deep white matter, without obvious foci of limited contusion. This is in contrast to focal TBI, where various stress responses and even death may be relatively confined. Diffuse injury can cause extensive axonal damage, which does not necessarily result in immediate cell death, but subsequent secondary damage including oxidative stress, ERS, and inflammatory cascades can crosstalk and progress gradually in the subacute as well as the chronic phase [13, 15]. Restricted by limited reports, it would be of great interest to explore the heterogeneity of ERS/UPR cascades in the context of different conditions of TBI.

We are also interested in the UPR in different cell types following TBI. In 2018, Hood et al. showed that ERS was activated in neonatal hippocampal neurons after TBI and that both neuronal loss and one-trial contextual fear memory were improved in CHOP knockout mice [211]. It is worth noting that the knockout mice involved all cell types, so caution may be warranted regarding the interpretation of the functional improvement. In 2016, the work of Chen et al. demonstrated that the Ca^2+^-dependent phosphatase, calcineurin-β (CNβ) was induced in astrocytes after TBI [212]. CNβ directly interacted with PERK, promoting its dimerization/oligomerization and autophosphorylation. Furthermore, activation of the CN-PERK-eIF2α pathway correlated with GFAP expression, suggesting this pathway may also directly regulate reactive gliogenesis signaling. Subsequent in vitro studies have shown that overexpression of CNβ in astrocytes is neuroprotective. Notably, the CNβ-PERK interaction was not assessed in the TBI model, which needs to be complemented in follow-up work. Most of the studies intervened untargeted in the whole brain, which potentially affects ERS in all cell types. In vitro cell culture or in vivo application of conditional knockout mice will help to assess this topic.

## Management of TBI through modulation of ERS

Although the mechanisms underlying the involvement of ERS in pathological progression after TBI are not fully understood, attempts to target ERS to manage TBI are still emerging. Current strategies can be summarized in two ways: the first is the regulation of crucial effectors in the ERS pathway by genetic means (CHOP, Sig-1R, etc.), and the other is to modulate ERS with specific compounds by pharmacological methods (Table 2). Relevant studies have been mainly conducted based on pharmacological tools. Given the complex cascade of responses following TBI and the crosstalk between multiple mechanisms, interventions targeting various mechanisms, including anti-ERS, through different means may offer more effective neuroprotection, as indicated by several studies [125, 201]. These studies are still in the preclinical phase, and related work must advance rapidly to validate clinical applicability. In addition, there exist small molecule compounds or established drugs (KIRA6 [137] (inhibits the activation of IRE1) and metformin [192]) that have apparent anti-ER stress effects in other models but have yet to be explored in TBI models. Considering the definite presence of ERS following TBI, the potential protective effects of these drugs against TBI warrant further investigation.

**Table 2:** Pharmacological compounds relevant to ERS in TBI.

Study	Name	Changes in related proteins	Effects
Li et al. [43]	JWH133	Depressing the PERK/eIF2α/ATF4/CHOP pathway Increasing p- Akt Reducing p-PERK in microglia	Promoting microglia differentiation toward protective M2 phenotype Improving oligodendrocyte survival
Tan et al. [98]	Salubrinal	Reducing ROS and p-eIF2α Inhibiting CHOP and caspase12 Inhibiting the release of Cytc Decreasing the Bax/Bcl-2 ratio Activating caspase3	Inhibiting ERS to reduce apoptosis Inhibiting oxidative stress Restoring mitochondrial function to inhibit the activation of mitochondrial apoptotic pathways
Li et al. [99]	Polydatin	Reducing ROS Reducing p-PERK and p-ATF6 Inhibiting p38 phosphorylation and cleaved caspase9 and caspase3 activation	Inhibiting the expression of ER markers Inhibiting mitochondrial apoptosis
Deng et al. [124]	Naringenin	Reducing p-eIF2α, ATF4, and CHOP	Inhibiting ERS, oxidative stress and apoptotic
Begum et al. [224]	DHA	Reducing p-eIF2α, ATF4, and CHOP Reducing APP and p-Tau	Reducing ERS and cell death.
Sun et al. [221]	TUDCA	Inhibiting p-PERK, p-eIF2a, ATF4, Pten, caspase12 and CHOP Increasing p-Akt and the Bcl-2/Bax ratio	Abolishing ERS Improving neurological function and alleviating brain edema
Zhai et al. [225]	Dexmedetomidine	Activating Sig-1R Increasing GRP78 Decreasing CHOP, caspase3, and p-JNK	Partially inhibiting ERS-induced apoptosis

*PERK* protein kinase RNA-like ER kinase, *p-PERK* phosphorylated PERK, *eIF2α* eukaryotic translation initiation factor 2 alpha, *p-eIF2α* phosphorylated eIF2α, *ATF* activating transcription factor, *p-ATF6* phosphorylated ATF6, *CHOP* C/EBP-homologous protein, *Akt* protein kinase B, *p-Akt* phosphorylated Akt, *ROS* reactive oxygen species, *Cytc* cytochrome c, *ERS* endoplasmic reticulum stress, *DHA* docosahexaenoic acid, *APP* amyloid precursor protein, *p-Tau* phosphorylated tau, *TUDCA* tauroursodeoxycholic acid, *Pten* phosphatase and tensin homolog, *Sig-1R* sigma-1 receptor, *GRP78* glucose-regulated protein 78, *p-JNK* phosphorylated c-Jun N-terminal kinase.

Aralia elata, a medicinal plant used in traditional medicine in East Asian countries and prominently used in Russian/Soviet medicine, has been found to have potential therapeutic benefits. Studies have shown that preparations of Aralia elata decreased the expression of ERS-related markers such as GRP78, CHOP, caspase12, and JNK while simultaneously increasing the expression of phosphorylated signal transducer and activator of transcription 3 (p-STAT3) and the Bcl-2/Bax ratio. A clinical trial was conducted on patients with long-term TBI with specific symptoms. They were administered 2 ml of Aralia tincture standardized for aralosides (three times a day for 30 days). The results indicated significant improvements in work capacity, sleep, and appetite, with reduced adverse symptoms [213]. However, considering the complexity of the components of Aralia tincture preparations, detailed studies should be conducted to further explore the mechanism of action.

Last but not least, determining the appropriate time to intervene is crucial. In the initial stages of ERS, moderate activation of the UPR is favorable for survival. However, it is vital to prevent further UPR activation when the threshold transitions from pro-survival to pro-apoptosis. Therefore, extensive and in-depth exploration is required to clarify the optimal timing of the intervention considering the duration of drug action.

## Conclusion and perspectives

To summarize, numerous studies have validated the activation of ERS following TBI and its pivotal role in determining cell fate. The pathophysiology of TBI is a multifaceted process. Mild ERS can be a protective mechanism by engaging with antioxidant, anti-inflammatory, and anti-apoptotic signaling pathways, efficiently mitigating secondary injury. In contrast, chronic and severe ERS may trigger a proapoptotic program, resulting in functional deterioration through downstream components of the UPR. Clinical and preclinical evidence for the management of TBI targeting ERS is limited. Therefore, it is imperative to further investigate the prosurvival/proapoptotic threshold of ERS and its mechanisms after TBI. Moreover, the method of administration and safe doses of the relevant molecular inhibitors also remain to be elucidated by more extensive experiments. In conclusion, it is reasonable to posit that therapies addressing ERS hold significant promise as a viable avenue of treatment for TBI.

### Supplementary information

Schematic diagram of the mechanism of injury in TBI.

Supplemental Figure Legends
