# Supplementary figures and images for: Endoplasmic reticulum stress and the unfolded protein response: emerging regulators in progression of traumatic brain injury
Source: Cell Death Dis. 2024 Feb 20;15(2):156. doi: 10.1038/s41419-024-06515-x (PMC10879178; doi:10.1038/s41419-024-06515-x)

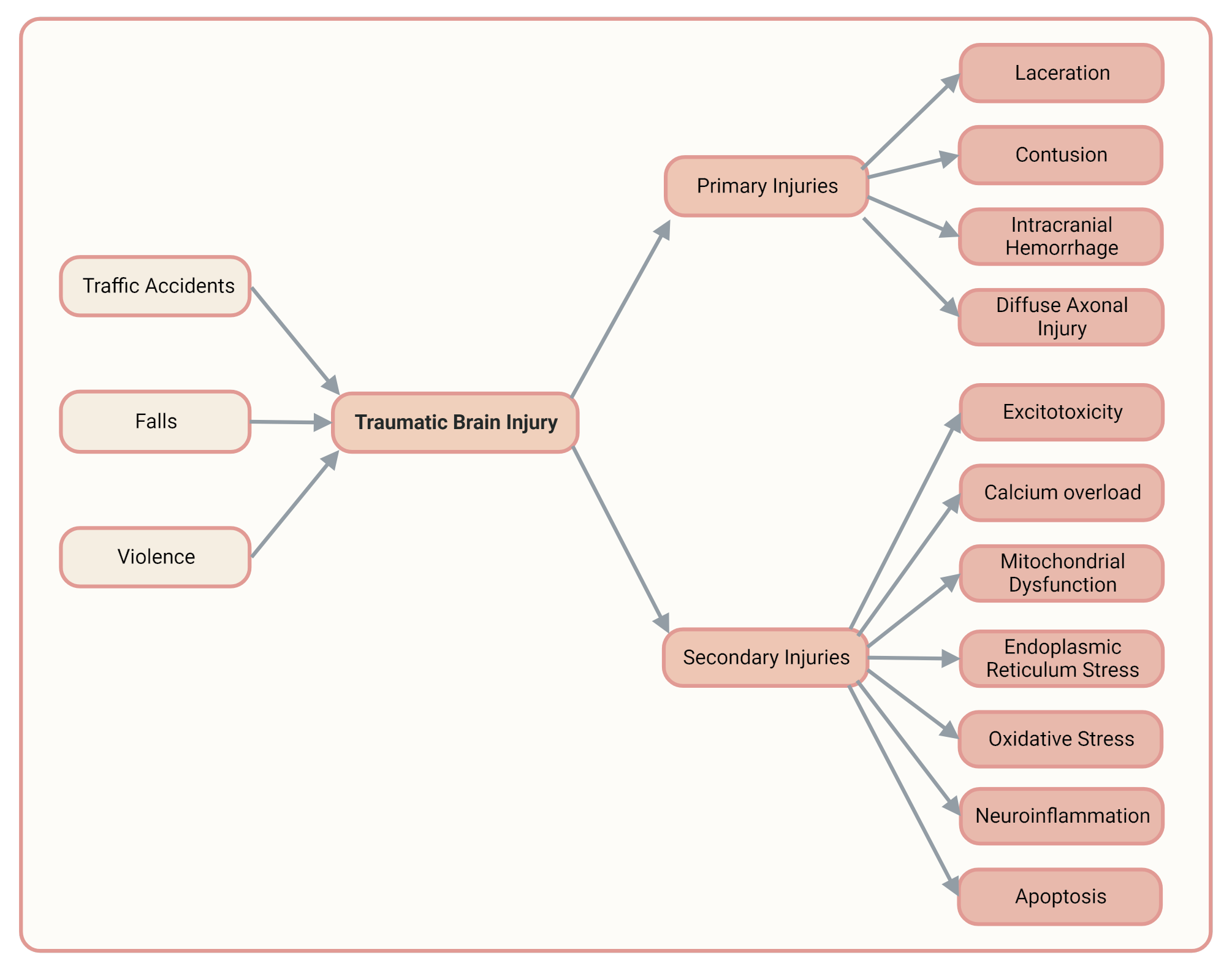

Supplement: Supplementary file 1 — Schematic diagram of the mechanism of injury in TBI. [file 41419_2024_6515_MOESM1_ESM.png]
