# Supplementary material for: Endoplasmic reticulum stress and the unfolded protein response: emerging regulators in progression of traumatic brain injury
Source: Cell Death Dis. 2024 Feb 20;15(2):156. doi: 10.1038/s41419-024-06515-x (PMC10879178; doi:10.1038/s41419-024-06515-x)
Supplement: Supplementary file 2 — Supplemental Figure Legends [file 41419_2024_6515_MOESM2_ESM.docx]

**Supplemental Figure Legends**

**Supplemental Figure S1.** Schematic diagram of the mechanism of injury in TBI. Created with BioRender.com.
